# Optical endomicroscopy and the road to real-time, in vivo pathology: present and future

**DOI:** 10.1186/1746-1596-7-98

**Published:** 2012-08-13

**Authors:** Charles S Carignan, Yukako Yagi

**Affiliations:** 1 NinePoint Medical, One Kendall Square B7501, Cambridge, MA 02139, USA; 2 Massachusetts General Hospital and Harvard Medical School, 101 Merrimac St. Suite 820, Boston, MA 02114, USA

**Keywords:** Barrett’s esophagus, Cancer, Confocal microscopy, Dysplasia, Endoscopy, In vivo imaging, Neoplasia, Optical coherence tomography, Optical imaging

## Abstract

Epithelial cancers account for substantial mortality and are an important public health concern. With the need for earlier detection and treatment of these malignancies, the ability to accurately detect precancerous lesions has an increasingly important role in controlling cancer incidence and mortality. New optical technologies are capable of identifying early pathology in tissues or organs in which cancer is known to develop through stages of dysplasia, including the esophagus, colon, pancreas, liver, bladder, and cervix. These diagnostic imaging advances, together as a field known as optical endomicroscopy, are based on confocal microscopy, spectroscopy-based imaging, and optical coherence tomography (OCT), and function as “optical biopsies,” enabling tissue pathology to be imaged in situ and in real time without the need to excise and process specimens as in conventional biopsy and histopathology. Optical biopsy techniques can acquire high-resolution, cross-sectional images of tissue structure on the micron scale through the use of endoscopes, catheters, laparoscopes, and needles. Since the inception of these technologies, dramatic technological advances in accuracy, speed, and functionality have been realized. The current paradigm of optical biopsy, or single-area, point-based images, is slowly shifting to more comprehensive microscopy of larger tracts of mucosa. With the development of Fourier-domain OCT, also known as optical frequency domain imaging or, more recently, volumetric laser endomicroscopy, comprehensive surveillance of the entire distal esophagus is now achievable at speeds that were not possible with conventional OCT technologies. Optical diagnostic technologies are emerging as clinically useful tools with the potential to set a new standard for real-time diagnosis. New imaging techniques enable visualization of high-resolution, cross-sectional images and offer the opportunity to guide biopsy, allowing maximal diagnostic yields and appropriate staging without the limitations and risks inherent with current random biopsy protocols. However, the ability of these techniques to achieve widespread adoption in clinical practice depends on future research designed to improve accuracy and allow real-time data transmission and storage, thereby linking pathology to the treating physician. These imaging advances are expected to eventually offer a see-and-treat paradigm, leading to improved patient care and potential cost reduction.

**Virtual Slides:**

The virtual slide(s) for this article can be found here: http://www.diagnosticpathology.diagnomx.eu/vs/5372548637202968

## Introduction

Cancers affecting the mucosal tracts are a substantial public health concern. Indeed, the incidence of esophageal adenocarcinoma (EAC) has increased dramatically in the United States [1,2] as well as most other Western developed societies [1]. The increased incidence is particularly alarming among US white men, which jumped 463% between 1975 and 2004 [2]; increases have also been observed in Europe, Australia, and New Zealand [3]. Age-standardized rates of EAC have increased up to 40% every 5 years in England and Wales [4], while annual increases in incidence rates of up to 5%, 5%, 6%, and 12% have been observed in Scotland, Scandinavia, France, and Switzerland, respectively [1,3,5,6]. EAC has a substantial impact on mortality, with a low 5-year survival rate (16.8%) [7]; overall, esophageal cancer has become the eighth most common cause of cancer death worldwide [1,3]. In contrast to esophageal cancer, the overall incidence rates of colorectal [8] and cervical cancers [9] have declined in the past several decades, but rates of gastric adenocarcinoma have remained relatively stable [10]. Despite these trends, colorectal cancer is still the third most common cancer worldwide, with the highest age-standardized incidence rates in Australia/New Zealand (45.7 per 100,000 men) and Western and Southern Europe (41.2 and 39.3 per 100,000 men, respectively) [11]. Colorectal cancer is the third leading cause of cancer mortality in men and women in the United States and accounts for 8% of all cancer deaths worldwide, with the highest mortality rates in Central and Eastern Europe [11]. Cervical cancer is the third most common cancer in women, with an estimated 530,000 new cases worldwide in 2008; incidence and mortality are lower in more developed areas such as Europe and North America than in developing countries in Africa and South America [11]. Gastric cancer is the fourth most common malignancy in the world (989,000 new cases occurring in 2008) and the second leading cause of cancer death (738,000 deaths worldwide), with the highest mortality rates in Eastern Asia and Central and Eastern Europe [11].

Given the incidence and mortality associated with epithelial cancers, effective strategies for early detection and treatment of premalignant lesions are essential. The benefits of early detection have been clearly demonstrated in cervical cancer, with population-based and cohort studies indicating that regular Pap screenings have decreased cervical cancer incidence and mortality by at least 80% [12]. Similarly, Barrett’s esophagus (BE) has been recognized as the premalignant lesion of EAC [13,14]. A growing number of studies have shown that regular endoscopic BE surveillance identifies patients with earlier stage cancer [15-17], leading to higher survival rates than more advanced disease [16]. Several retrospective studies have indicated that survival is prolonged if esophageal cancers are detected by endoscopic surveillance rather than by presenting symptoms [13,15,18].

This review discusses the substantial progress under way in endoscopic imaging, including the present state of technology, current approaches to imaging research, and the potential impact of these techniques on daily clinical practice in the near future.

### Paradigms in endoscopic biopsy: applications and limitations

Current approaches to endoscopic biopsy use external imaging, such as computed tomography (CT), magnetic resonance (MR), or white light endoscopy, to image suspect tissue. Despite advances in the field of endoscopic imaging, technical limitations of these modalities exist. These limitations may have important clinical implications, especially in optimizing cancer screening, diagnosis, and surveillance in the detection and histological assessment of premalignant lesions. For example, treatment guidelines for recognizing EAC and preventing mortality are largely based on endoscopic surveillance of patients with chronic, symptomatic gastroesophageal reflux disease and those with BE as well as use of histopathological assessment to evaluate the risk of BE progression to EAC [13,14,19]. Although currently considered the gold standard for surveillance [19], white light endoscopy is limited to the surface of the mucosa and depends on clinical changes to signify underlying disease. External sources (CT/MR) typically lack sufficient resolution to provide accurate guidance for biopsy location determination.

When BE is identified, targeted biopsies and four-quadrant, random biopsies are obtained to detect invisible neoplasias [14,19,20], but these strategies may be unreliable [21] because of sampling error and other practical limitations. When performed appropriately, a random sampling technique reduces the area of tissue surveyed, covering as little as 5% of the surface area of BE tissue [22]. Mucosal irregularities of early neoplasias are often discrete and easily missed during standard BE surveillance endoscopy [20]. In surgical resection specimens, up to 43% of patients with confirmed high-grade dysplasia had adenocarcinomas that were missed before surgery, despite the use of endoscopic biopsy [23]. Given the small amounts of histologically ambiguous tissue retrieved, the potential for diagnostic misinterpretation and variability among pathologists is considerable, a problem that has been demonstrated in several studies [22,24-26]. The time delay between endoscopy and diagnosis is another limitation, with separate procedures required for the detection and treatment of dysplasia [26]. The current biopsy approach is uncomfortable and time consuming for patients, often requiring a lengthy period of sedation and posing risks of bleeding and perforation [20,27]. The limitations of current imaging and biopsy methods represent an unmet need in the early detection of mucosal dysplasias.

### Current and investigational technologies for in vivo imaging

Unlike current techniques, newly developed in vivo imaging technologies offer the potential to guide biopsy and to move toward real-time pathology. These tools may enable immediate optical histology of the mucosal layer during ongoing endoscopy, or *virtual histology*, allowing visualization of living cells and cellular structure at and below the mucosal surface [28]. Compared with conventional radiologic and endoscopic techniques, these newer technologies achieve higher-resolution microscopic images with wider-ranging visualization of the target tissue (Table 1).

**Table 1:** Comparison of current and investigational imaging technologies

	**Radiology**			**Endoscopy**	**Endomicroscopy**	
Resolution	1 cm	1 mm	100 μm	~100 μm	10 μm	1 μm
Field of view	50+ cm	30+ cm	2–5 cm	140°	3 mm	0.3 mm
Technology	Radio nucleotide, DOT, PET	MRI, CT, US	EUS, IVMRI, X-ray	Standard and high-definition video endoscopes	OFDI, OCT	SECM, Micro OCT, FFOCM
						
	**Organ**	**Organ**	**Organ**	**Tissue surface**	**Architectural**	**Cellular**
	Thallium	MRI	US	White light	OCT*	CM

*Volumetric.

CM = confocal microscopy; CT = computed tomography; EUS = endoscopic ultrasound; IV = intravenous; FFOCM = full-field optical coherence microscopy; MRI = magnetic resonance imaging; OCT = optical coherence tomography; OFDI = optical frequency domain imaging; PET = positive emission tomography; SECM = spectrally encoded confocal microscopy; US = ultrasound.

### Confocal laser endomicroscopy

Confocal laser endomicroscopy (CLE), a recent endoscopic advance, allows real-time high-resolution histologic analysis of targeted tissue during endoscopy [29]. The CLE illuminates tissue with a low-powered laser focused by an objective lens into a single point within a fluorescent specimen [30,31]. A confocal microscope is used to exclude light above and below a plane of interest, thus allowing for an optical section to be observed, similar to a histologic tissue section [29]. The generated gray scale image represents one focal plane within the examined specimen [31]. The mucosa typically can be imaged to a depth of 100 to 150 μm with this technique [22].

**Figure 1:**
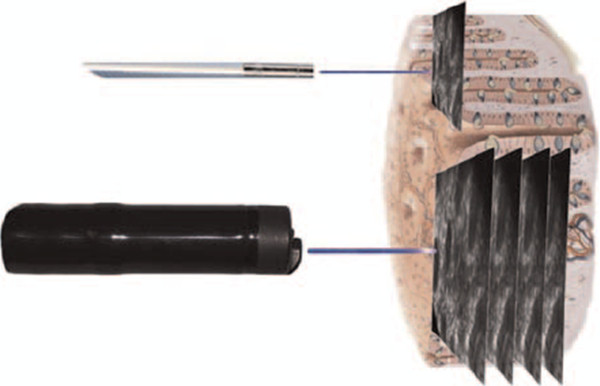
**Two types of confocal endomicroscopy systems are currently available in the United States.** A mini-probe based system (MaunaKeaTechnology, France; upper panel) can be used through the working channel of most conventional endoscopes. In the confocal laser endomicroscope (Pentax, Japan; lower panel), the laser scanner is integrated into the endoscope. Reprinted with permission from Goetz M, Kiesslich R [33].

Currently, two devices are available and have received the CE Mark for use for CLE [29,32] (Figure 1[33]), and a third is under development. The endoscope-based CLE (eCLE; Cellvizio®, Pentax Corporation, Montvale, NJ, USA, and Tokyo, Japan) uses a confocal fluorescence microscope integrated into the distal tip of a conventional upper endoscope or colonoscope [29,30]. The probe-based CLE (pCLE; Mauna Kea Technologies, Newtown, PA, USA, and Paris, France) uses a fiber-optic probe bundle with a laser microscope inserted through the accessory channel of a standard endoscope [29,30]. Although lateral and axial resolution is better with eCLE than with pCLE, the eCLE is considerably bulkier [29]. The pCLE is more useful in smaller spaces [29]; recent data demonstrated the feasibility of using pCLE for visualization of intra-abdominal organs, including liver, pancreas, spleen, and lymph nodes in a porcine model [34]. Development of a probe-based volumetric CLE device is under way.

**Figure 2:**
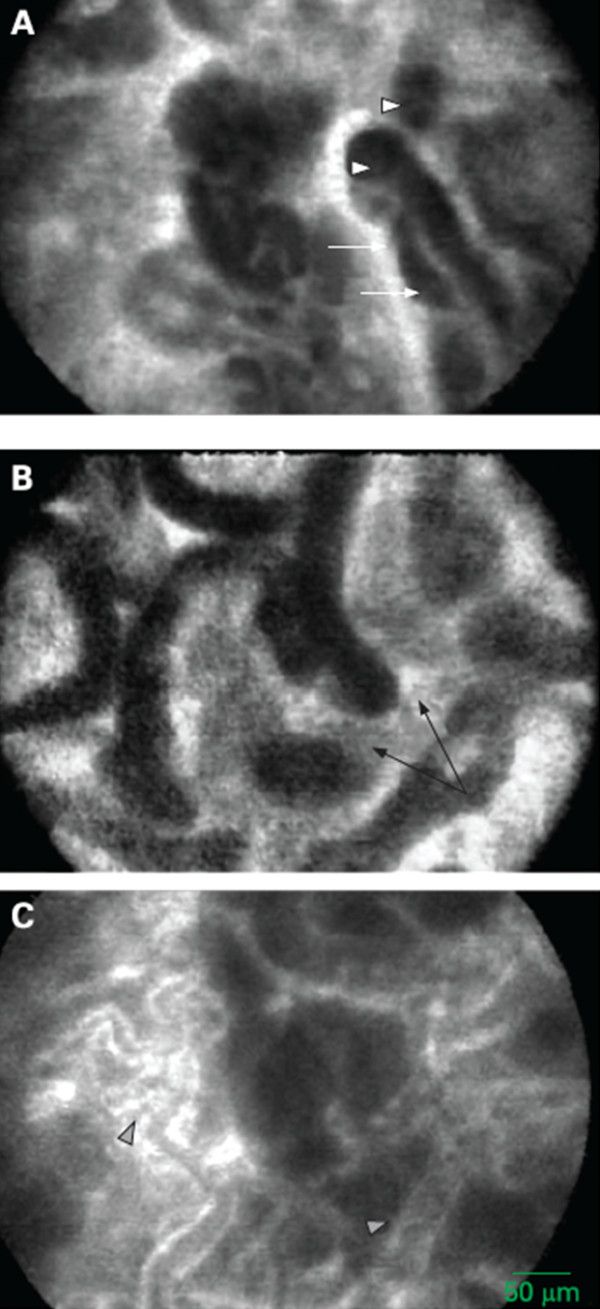
**Barrett’s mucosa with early mucosal adenocarcinoma recorded with in vivo miniprobe confocal laser microscopy.** Neoplastic characteristics include irregular epithelial lining with variable width (white arrows), increased cell density seen as dark areas with variable fluorescein uptake (white triangle), fusion of glands (black arrow), and irregular dilated blood vessels (arrowheads). Reprinted with permission from Pohl H, et al. [37].

**Figure 3:**
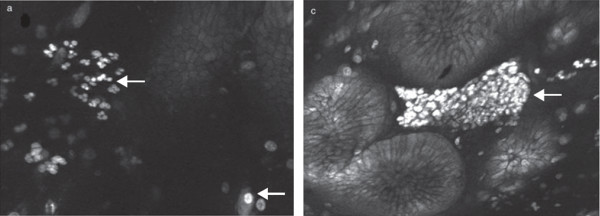
**Neutrophils and microabscesses of *H. pylori*-positive gastric mucosa. (A)** Neutrophils were identified by their nuclear features. White arrow shows the mononuclear cell. **(B)** Microabscesses appeared in superficial epithelium and foveola. Reprinted with permission from Ji R, et al. [43].

Since 2004 when confocal endomicroscopy was first used for diagnosing colorectal pathology [35], CLE has shown promise in a number of clinical applications. Indeed, CLE potentially may be used in the same manner as endoscopic biopsy [36]. Both eCLE and pCLE have had high accuracy (≥90%) in diagnosing BE and Barrett’s-associated neoplastic changes (Figure 2) [37,38]. CLE also can detect lymphocytic and collagenous colitis in chronic diarrhea [39,40], identify the microarchitecture of early gastric cancer [41,42], detect *Helicobacter pylori* infection with high accuracy (Figure 3) [43], and detect villous atrophy in celiac disease [44]. Preliminary data have shown that CLE can detect malignant changes in pancreatic tissue [45] and premalignant changes in peripheral lung nodules [46], urothelium [47], and cervical epithelium [48].

Although commercially available, the place of CLE in current diagnostic paradigms versus a conventional histopathological examination is still evolving [30]. With appropriate contrast agents, CLE has the potential for subcellular resolution, reducing the number of biopsies required [29], as well as for molecular characterization [49]. However, available CLE devices have a narrow field of view and cannot penetrate beyond the mucosa, allowing visualization of only superficial mucosal layers [29,30]. Moreover, CLE does not provide an archive of tissue for full molecular characterization [29], and contrast agents can limit the procedure duration and ability to obtain repeat images [22].

### Spectrally encoded confocal microscopy

**Figure 4:**
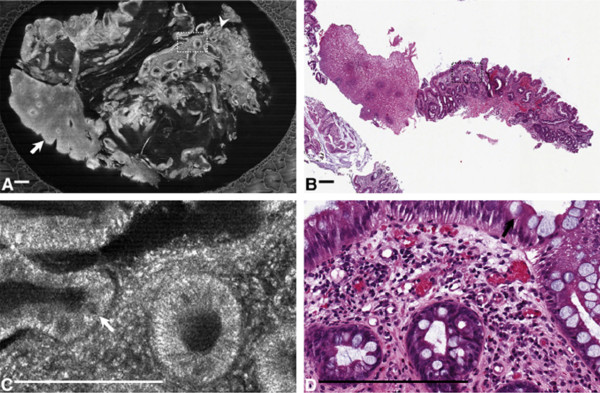
**SECM and histopathological images of BE stained with 0.6% acetic acid. (A)** Large-area SECM image shows columnar epithelium (*arrowhead*) and squamous epithelium (*arrow*). **(B)** Histopathologic image demonstrates squamoglandular junctional mucosa. **(C)** High-magnification SECM image shows the presence of goblet cells (*arrow*). **(D)** High-magnification histopathological image shows BE with the presence of goblet cells (*arrow*). Scale bars represent 250 μm. Reprinted with permission from Kang D, et al. [50].

Spectrally encoded confocal microscopy (SECM) is a high-speed technique based on reflectance imaging technology [50]. This method couples broadband or wavelength-swept narrowband light into a single optical fiber, which then illuminates a transmission grating and objective lens at the end of the confocal probe to encode one-dimensional spatial information reflected from a sample [50-52]. Because SECM detects spatial information externally to the probe, it can obtain highly detailed images at very high speeds (up to 10 times faster than the video rate), while the size of the optics is small enough to be incorporated into a small-diameter catheter or endoscope [50,51]. The SECM allows for large field confocal images without the need for contrast agent and may permit the imaging of extended areas of tissue [50,51,53]. Given that SECM can achieve, in principle, comprehensive confocal endomicroscopy of the entire distal esophagus, this technology is being investigated for imaging upper gastrointestinal (GI) tissues [50]. Preliminary assessment indicates that SECM can reveal the architectural and cellular features of gastroesophageal tissues, including the presence of goblet cells, columnar epithelium, and squamous epithelium in BE (Figure 4) [50,52]. A recent study in eosinophilic esophagitis showed that SECM of biopsy samples was functional in accurately providing eosinophil counts, as well as in identifying microscopic abnormalities such as abscess, degranulation, and basal cell hyperplasia [54].

### Spectroscopy-based imaging

**Figure 5:**
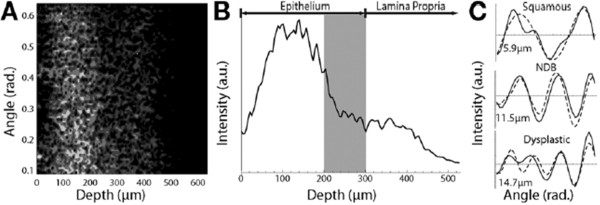
**Comparison of depth, area, and images achieved with a/LCI and confocal microscopy.** Typical a/LCI data. **(A)** Angle-resolved depth scan of light scattered from tissue. Lighter shades of gray indicate increased amount of scattered light. **(B)** Amplitude scan indicating depth increments used for processing. Tissue layers are labeled, and *gray bar* indicates basal layer (optical coherence tomography). Example angular scans for 3 tissue types pictured (solid line) with best-fit Mie theory solutions (dashed line) and size indicated. Reprinted with permission from Terry NG, et al. [26].

Angle-resolved low coherence interferometry (a/LCI), a light-scattering technique, identifies early dysplasia based on nuclear diameter differences [22,26,55]. This method measures the angular distribution of scattered light as a function of depth beneath the tissue surface [26] and achieves depth resolution through a process similar to that used in optical coherence tomography (OCT) [22,26]. The a/LCI device can assess nuclear size at multiple depths [22], with deeper penetration than confocal microscopy approaches (up to 200–300 μm of the epithelial tissue layer compared with the surface and uppermost 100 μm of tissue with endoscopic confocal microscopy) [26,55]. The a/LCI data are analyzed and reported according to a best-fit analysis (Figure 5), with nuclear measurements in cell and tissue types reported with an accuracy of 0.2 to 0.3 μm [22]. The a/LCI device can provide instant high-resolution images non-invasively without the need for image interpretation by an endoscopist or administration of contrast agents [26].

Recent clinical studies have explored a/LCI in the assessment of dysplasia in esophageal [26] and intestinal [55] tissues. In the first in vivo clinical study of a/LCI, 46 patients undergoing routine endoscopic surveillance for BE were scanned with the a/LCI system and the results correlated with an endoscopic biopsy specimen [26]. The nuclear size measurements generated for deep epithelial tissue (200–300 μm beneath the surface) separated dysplastic from non-dysplastic tissue with an accuracy of 86%, using a cutoff of 11.84 μm to separate the two types [26]. Using this same cutoff, a/LCI distinguished dysplastic BE specimens from indeterminate and non-dysplastic BE with a sensitivity of 100% (13/13; 95% confidence interval [CI], 0.75–100) and a specificity of 85% (76/89; 95% CI, 0.76–0.92) [26]. Similarly, a pilot ex vivo study of 27 patients undergoing partial colonic resection demonstrated high diagnostic value of this method at a depth 200 to 300 μm beneath the mucosal surface, with a/LCI separating dysplastic from healthy intestinal tissues with a sensitivity of 92.9%, a specificity of 83.6%, and an overall accuracy of 85.2% [55].

Several other spectroscopy-based imaging techniques are under investigation in various clinical applications. Laser-induced fluorescence is a technique based on the principle that certain compounds exhibit a characteristic fluorescence emission when excited by light [56]. This technology has been shown to detect malignant colonic tissue [57] and to distinguish malignant tissue from metaplastic and normal tissue in BE [56,58]. Multimodal hyperspectroscopy is based on tissue fluorescence and reflected light measurements, which are analyzed with computed-based algorithms to differentiate between abnormal and normal tissues [59]. Although more extensively explored for use in detecting cervical cancer [59,60], clinical studies in BE patients are under way [61].

### Optical coherence tomography

OCT is an imaging technique first introduced for use in biological tissues in 1991 [62] that generates high-resolution, cross-sectional, subsurface images by using low-coherence interferometry to measure the echo time delay and intensity of back-scattered light [63]. OCT is analogous to ultrasonography, except that OCT measures the intensity of infrared light rather than sound waves [64]. With OCT, depth intensity is measured by time-domain measurements, allowing for image construction for all three dimensions.

**Figure 6:**
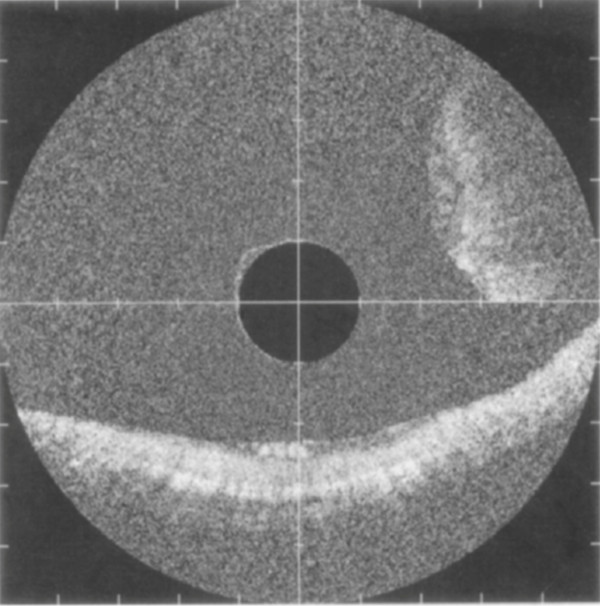
**OCT image of colon adenoma (2-o’clock position).** A well-organized linear crypt pattern is not present and image is darker because of altered light scattering compared with the nondysplastic mucosa as seen in the normal mucosa running horizontally in the 6-o’clock position. The marks of the vertical and horizontal axes are 1 mm apart. Reprinted with permission from Pfau PR, et al. [74].

**Figure 7:**
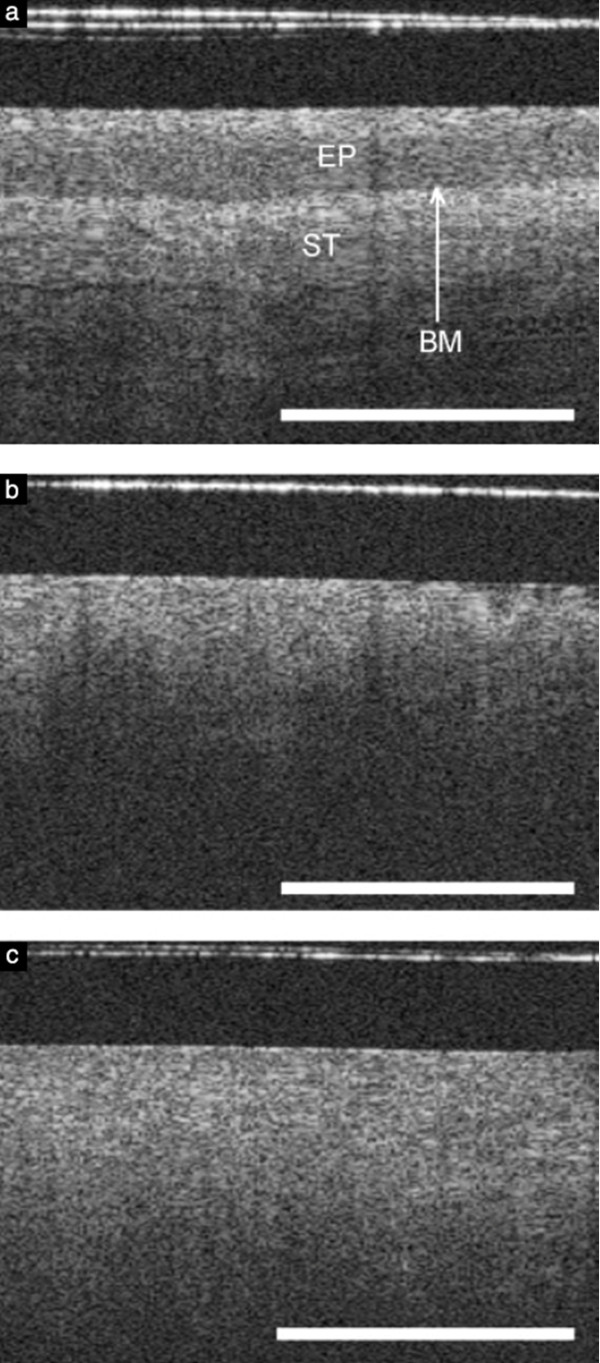
**Optical coherence tomography (OCT) images. (A)** OCT image of normal cervical tissue, showing a well-organized, three-layer architecture (optical structure) with sharp borders. The thin basement membrane (BM) could not be resolved by OCT. However, because the basement membrane separates the epithelium (EP) from stroma (ST), a sharp interface could be visualized (length of the white bar: 1 mm). **(B)** OCT image showing a cervical intraepithelial neoplasia (CIN-3) lesion. The intensity of the stromal layer increases with less-organized layer architecture. The stroma seemed to push its way towards the surface as vertical columns. **(C)** OCT image showing invasive carcinoma. The tissue surface is an unstructured homogeneous highly backscattering region with a complete lack of layer architecture (optical structure). The basement membrane is no longer intact or defined and the tissue microstructure is no longer organized. Reprinted with permission from Gallwas J, et al. [76].

Since its use was first described in ophthalmology to image the transparent structures of the anterior eye and retina [65], OCT has evolved to include a wide spectrum of clinical applications. The successful use of OCT imaging techniques has been described in many biologic tissues, including human coronary arteries [66,67]; esophageal [68-71], gastric [72,73], and intestinal [74] tissues (Figure 6); pancreatic and biliary tissues [75]; cervical epithelium (Figure 7) [76]; and urologic tissues [77]. Extensively studied in GI applications [72,74,78], OCT has shown accuracy in diagnosing specialized intestinal metaplasia in BE with a sensitivity of 81% [71,79].

Several OCT systems are currently in use or under investigation. The original OCT technology, now called time-domain OCT (Niris®, Imalux Corporation, Cleveland, OH, USA) [80,81], has been described in detail elsewhere [64,78]. Interferometric synthetic aperture microscopy uses computed imaging and synthetic aperture techniques to modify OCT signals to achieve three-dimensional, spatially invariant resolution for all depths in a cross-sectional scan [82-84]. The feasibility of using this technology to image human breast tissue has recently been demonstrated [83,84].

Despite the diagnostic potential of time-domain OCT, its relatively slow imaging speed has precluded its ability to survey large areas of the GI tract, limiting its use to point-sampling with a field of view comparable to that of conventional biopsy [70,85]. However, a new technologic approach to OCT allows dramatic increases in imaging speed without compromising image resolution or quality [70,86-88]. This technology, referred to as Fourier-domain OCT [81] or optical frequency domain imaging [70], is also called volumetric laser endomicroscopy (VLE). VLE acquires cross-sectional images by using a focused, narrow-diameter beam to repeatedly measure the delay of reflections from within the tissue sample [70]. Interferometry is used to measure the delay intervals, while Fourier transformation is used to compute traditional A-lines, or depth scans, which comprise the tissue reflectivity as a function of depth along the beam. Unlike time-domain OCT, VLE uses a fixed wavelength or swept-source technology in which the wavelength of a monochromatic light source is rapidly scanned to measure the interference signal as a function of wavelength [70,87].

The use of a balloon-based VLE system with helically scanning optics for esophageal imaging has been described [68-70,85]. With this system, the optical components of the catheter are positioned with the esophageal lumen via a balloon-centered probe [70,85]. After the balloon is inflated, the distal esophagus is dilated and the imaging optics become centered. Optics are slowly pulled back during the imaging procedure while the imaging optics are rotated by a probe scanner; thus, the entire portion of the esophageal lumen that was in contact with the balloon is scanned in a helical or circumferential fashion [70]. Real-time, volumetric images are obtained by scanning the imaging beam over the tissue surface in two dimensions [69].

**Figure 8:**
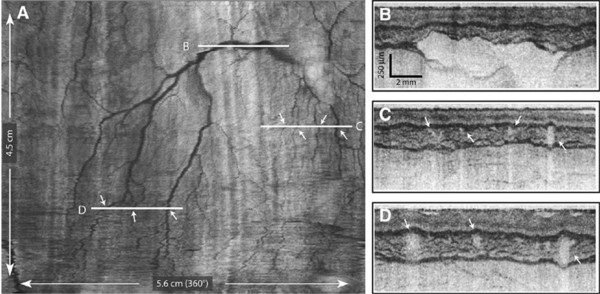
**High-resolution images from VLE. (A)** A comprehensive vascular map derived from the structural image set. **(B–D)** Cross-sectional images at the indicated locations. Arrows indicate corresponding vessels in the vascular map and cross-sectional images. Reprinted with permission from Vakoc BJ, et al. [70].

**Figure 9:**
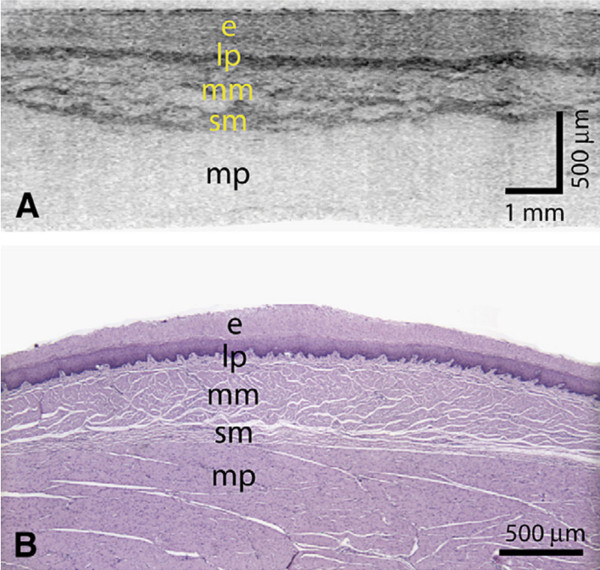
**High-resolution images from VLE. (A)** A transverse cross-sectional image showing all architectural layers of the squamous mucosa, including the epithelium (e), lamina propria (lp), muscularis mucosa (mm), submucosa (sm), and muscularis propria (mp); because of the large change in esophageal circumference during imaging (56 mm) and after resection (~22 mm), the cross-sectional image is displayed over a proportionately larger width. **(B)** Representative histology from the same swine (H&E, orig. mag. *x*2). Reprinted with permission from Vakoc BJ, et al. [70].

**Figure 10:**
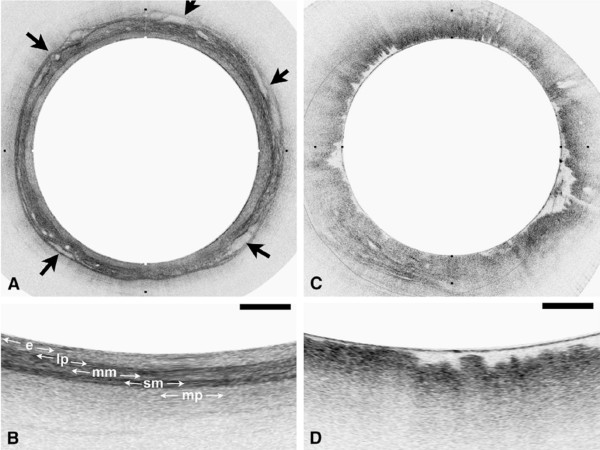
**OFDI images obtained from patients with a normal-appearing stomach and esophagus by endoscopy. (A)** OFDI image of squamous mucosa. **(B)** Expanded view of A demonstrates a layered appearance, including the epithelium (e), lamina propria (lp), muscularis mucosa (mm), submucosa (sm), and muscularis propria (mp). Vessels are clearly identified in the submucosa (arrows). **(C)** OFDI image of gastric cardia. **(D)** Expanded view of C demonstrates vertical pit and crypts, regular, broad architecture, high surface backscattering, and diminished image penetration. Tick marks in **A** and **C** and scale bars in **B** and **D** represent 1 mm. Reprinted with permission from Suter MJ, et al. [68].

**Figure 11:**
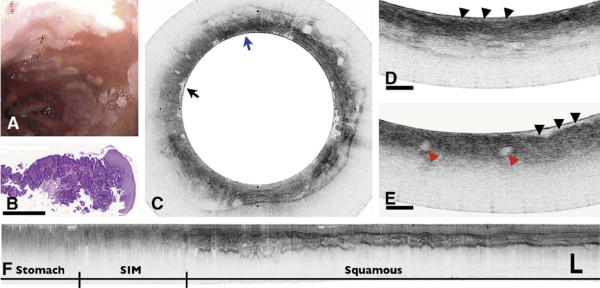
**Barrett’s esophagus with dysplasia. (A)** Videoendoscopic image reveals a patchy mucosa consistent with SIM. **(B)** Histopathologic image of the biopsy specimen taken from the SCJ demonstrates intestinal metaplasia and low-grade dysplasia (H&E, orig. mag. °–2). **(C)** Cross-sectional OFDI image demonstrating regions consistent with SIM without dysplasia *(blue arrow)* and specialized intestinal metaplasia with high grade dysplasia *(black arrow)*. **(D)** Expanded view of **C** taken from the region denoted by the blue arrow in **C,** demonstrating good surface maturation *(arrowheads)*, which is consistent with SIM without dysplasia. **(E)** Expanded view of **C** taken from the region denoted by the *black arrow* in **C,** demonstrating features consistent with high grade dysplasia, including poor surface maturation *(black arrowheads)* and the presence of dilated glands *(red arrowheads)* in the mucosa. **(F)** A longitudinal slice highlights the transition from gastric cardia, through a 9-mm segment of specialized intestinal metaplasia and finally into squamous mucosa. Scale bars and tick marks represent 1 mm. Reprinted with permission from Suter MJ, et al. [68].

Preliminary data for the VLE system have shown its ability to image the entire distal esophagus at a higher speed and greater sensitivity compared with time-domain OCT [70,85]. VLE enables full-length surveillance of target areas with a combination of resolution and depth of surface penetration (3-mm penetration, <10-μm resolution depth) [52]. When used in swine models, VLE provided high-resolution images of the anatomic layers and vasculature from the distal esophagus and gastroesophageal junction (Figures 8 and 9) [70]. In the first clinical experience with this technique, VLE successfully imaged the microscopic architecture of the distal esophagus in 10 of 12 patients undergoing routine esophagoduodenostomy for BE screening and surveillance (Figures 10 and 11), with volumetric images acquired in less than 2 minutes [68]. Most recently, the feasibility of VLE-guided biopsy with laser marking was demonstrated in swine esophagus, a strategy with the potential to increase the diagnostic accuracy of current surveillance protocols and to guide interventional treatments [69].

### Roles and impact of the advances in optical biopsy

In vivo pathology imaging devices and the rapid evolution of the technology have the potential to make real-time diagnosis the new standard, with immediate diagnosis and management during endoscopy. The new optical biopsy technologies provide better quality, detailed, high-resolution images and allow visualization of living cells and cellular structures at and below the mucosal surface during ongoing endoscopy [28,35]. The convergence of imaging and pathology may provide distinct advantages in cancer detection and diagnosis without the limitations and risks inherent with biopsy procedures. With these technologies, maximal diagnostic yields may be obtained, leading to appropriate staging through guided biopsy while minimizing the frequency and error potential of random biopsy protocols. In vivo cellular information can be delivered before biopsies are performed, or imaging files may be transmitted with biopsies, potentially improving the efficiency and accuracy of diagnosis.

Despite the potential these techniques may offer to standard clinical practice, barriers remain. Optical biopsy techniques can identify neoplastic changes in a variety of biologic tissues, but prospective studies in large cohorts are needed to establish concrete sensitivity and specificity of the respective technologies, in each target organ, versus the need for biopsy. To achieve widespread clinical adoption, these technologies must be accurate, efficient for use in the endoscopic setting, reliable, user-friendly, patient-friendly, and cost-effective [22,89]. Wide acceptance and interpretation capabilities, which require comprehensive physician education and training, are also necessary to establish appropriate comfort with use. Investigators are currently working to improve the accuracy, speed, and ease of interpretation of these technologies [89]. In addition, research is under way to allow real-time data transmission and storage, thereby linking pathology results to the treating physician.

## Conclusion

As epithelial malignancies move toward earlier detection and treatment, the ability to accurately detect precancerous lesions has an increasingly important role in controlling cancer incidence and mortality. With new optical techniques, high-resolution images of early neoplastic changes in various tissues and organs can now be captured in real time through endoscopes, catheters, laparoscopes, and needles [78]. Although the diagnostic potential of these technologies is rapidly expanding, their clinical adoption will depend on present and future research demonstrating improved imaging performance and functionality, and the development and acceptance of new guidelines for imaging [78]. Novel optical imaging technology offers the opportunity to utilize a see-and-treat paradigm, potentially leading to improved patient care and cost reduction.

## Abbreviations

a/LCI: Angle-resolved low-coherence interferometry; BE: Barrett’s esophagus; CI: Confidence interval; CLE: Confocal laser endomicroscopy; CT: Computed tomography; EAC: Esophageal adenocarcinoma; eCLE: Endoscope-based confocal laser endomicroscopy; GI: Gastrointestinal; MR: Magnetic resonance; OCT: Optical coherence tomography; pCLE: Probe-based confocal laser endomicroscopy; SECM: Spectrally encoded confocal microscopy; VLE: Volumetric laser endomicroscopy.

## Competing interests

CSC is an employee of NinePoint Medical. YY has no competing interests to declare.

## Authors’ contributions

CSC and YY made substantial contributions to the conception and design of this review and were involved in drafting the manuscript or revising it critically for important intellectual content. Both authors have given final approval of the version to be published.
